# Structural Insights into Mechanisms Underlying Mitochondrial and Bacterial Cytochrome c Synthases

**DOI:** 10.3390/biom14121483

**Published:** 2024-11-21

**Authors:** Pema L. Childs, Ethan P. Lowder, Deanna L. Mendez, Shalon E. Babbitt, Amidala Martinie, Jonathan Q. Huynh, Robert G. Kranz

**Affiliations:** Department of Biology, Washington University, St. Louis, MO 63146, USA; c.pema@wustl.edu (P.L.C.); ethanlowder@hms.harvard.edu (E.P.L.); dlmendez@wustl.edu (D.L.M.); sebabbit@wustl.edu (S.E.B.); amidala@wustl.edu (A.M.); h.jonathan@wustl.edu (J.Q.H.)

**Keywords:** heme, cytochrome c, heme attachment, cytochrome c synthase, structure, mechanisms, respiration, heme metabolism, MLS

## Abstract

Mitochondrial holocytochrome c synthase (HCCS) is an essential protein in assembling cytochrome c (cyt c) of the electron transport system. HCCS binds heme and covalently attaches the two vinyls of heme to two cysteine thiols of the cyt c CXXCH motif. Human HCCS recognizes both cyt c and cytochrome c_1_ of complex III (cytochrome bc_1_). HCCS is mutated in some human diseases and it has been investigated recombinantly by mutational, biochemical, and reconstitution studies in the past decade. Here, we employ structural prediction programs (e.g., AlphaFold 3) on HCCS and its two substrates, heme and cytochrome c. The results, when combined with spectroscopic and functional analyses of HCCS and variants, provide insights into the structural basis for heme binding, apocyt c binding, covalent attachment, and release of the holocyt c product. Results from in vitro reconstitution of purified human HCCS using cyt c and cyt c_1_ peptides as acceptors are consistent with the structural modeling of substrate binding. Reconstitution of HCCS and cyt c_1_ provides an approach to studying cyt c_1_ assembly, which has been refractile to recombinant in vivo reconstitution (unlike HCCS and cyt c). We propose a structural basis for release of the holocyt c product from HCCS based on in vitro studies and on cryoEM structures of the bacterial cyt c synthase (CcsBA) active site. We analyze the kinetoplastid mitochondrial synthase (KCCS), and hypothesize a molecular evolutionary path from mitochondrial endosymbiosis to the current HCCS.

## 1. Introduction

Nearly all organisms have c-type cytochromes, heme proteins involved in many respiratory and photosynthetic electron transport chains. Cytochromes c are unique in that heme is covalently attached to the protein, typically by two thioether bonds. Cysteines of a CysXxxXxxCysHis (CXXCH) heme binding motif in cytochrome c (cyt c) attach to the two vinyl groups in heme. The first thiol (in CXXCH) is attached to the 2-vinyl and the second thiol to 4-vinyl, and thus heme is regio-specifically positioned. Three pathways occur in nature for cyt c biogenesis, called Systems I, II, and III [1,2,3,4,5,6]. Systems I and II are present in prokaryotes and likely emerged between two and three billion years ago. They comprise dedicated membrane proteins that deliver the heme to the periplasm and attach it to many different c-type cytochromes that evolved in bacteria. All c-type cytochromes in bacteria contain an SEC-dependent signal sequence to direct the apoprotein to the periplasm for heme attachment. Systems I and II end with a cyt c synthase, called CcmF/H for System I and CcsBA for System II. Cyt c synthases attach the heme to the apocyt c substrate, and thus each has two substrates that bind at the active site.

System III evolved in mitochondria less than a billion years ago [3,5]. It is composed of a single enzyme called holocyt c synthase, HCCS (Supplementary Figure S1). HCCS, sometimes called CCHL (for cyt c heme lyase), binds heme in the mitochondrial intermembrane space and attaches it to cyt c. Some mitochondria retained System I in evolution, but most eukaryotes use HCCS (Supplementary Figure S2). HCCS was discovered in *Saccharomyces cerevisiae* as a gene required for attachment of heme to cyt c [7]. A related enzyme, HCC_1_S in *S. cerevisiae*, is used for assembly of cyt c_1_ of complex III (cyt bc_1_) [8]. In mammals, a single HCCS attaches heme to both cyt c and cyt c_1_ [9]. The high sequence identity between HCCS and HCC1S suggests that the structures and mechanisms will be similar. Indeed, Hamel and colleagues have shown that HCCS and HCC_1_S are interchangeable in function under certain conditions such as overexpression [6,9]. A particular interest in human HCCS stems from its defects that result in the disease called microphthalmia with linear skin defects (MLS) [10,11,12,13]. Since the human HCCS gene resides on the X chromosome and HCCS is a required protein, only females have MLS, with one wt HCCS allele and another allele with an HCCS (MLS) mutation. HCCS E159K and R217C are common mutations associated with MLS [14,15]. Insights into these defects from structural and biochemical perspectives are described here.

Previous studies on HCCS have taken advantage of the ability to functionally express human HCCS and cyt c in *Escherichia coli* and to purify GST-tagged HCCS, with and without its substrates, heme and cyt c [16,17,18,19,20]. These studies have led to a better understanding of key residues and identification of four possible domains in HCCS. For example, His154 forms an axial ligand to heme, and HCCS H154A variants do not bind heme in vivo or in vitro. HCCS His154 is found in a region termed domain II, here called the His154 helix, which is proposed to be part of the heme binding site (Supplementary Figure S1A). Because cyt c is not co-purified with the HCCS H154A variant, unlike wt HCCS, heme binding was proposed as the first step in a four-step mechanism for HCCS activity [3,16]. Critical to further studies on HCCS is a structural understanding of its active site for binding heme (step 1), then cyt c (step 2), thioether attachment (step 3), and release (step 4) of the holocyt c product.

We recently developed in vitro reconstitution with purified HCCS, where heme is attached to both cyt c and peptides containing CXXCH [21]. A minimal peptide substrate of 16 residues, to which heme was attached, included the CXXCH motif and the adjacent alpha helix-1 (sometimes referred to as the N-terminal alpha helix in cyt c). In vivo genetic results on the cyt c alpha helix-1 have also suggested that this region is important for biogenesis by HCCS [16,20,22,23,24,25,26]. Here we use in vitro studies to analyze human HCCS and its recognition requirements for cyt c_1_. The results are consistent with AlphaFold 3 (AF3) structural predictions on substrate binding sites.

A wealth of three-dimensional structures for System I and II have been published recently, with new insights into the structural bases for heme transport and attachment [27,28,29,30,31,32]. However, HCCS has been refractile to structural elucidation and remains a gap in the field. Because biochemistry and spectroscopy on recombinant HCCS enzymes has been informative [16,17,18,19,20,33], we here use recent structural prediction platforms like AF3 [34,35] and RoseTTAFold [36] on HCCS, and evaluate the active site in the context of information from spectroscopic and other approaches. We describe new analyses on CryoEM structures [28] of the bacterial cyt c synthase, CcsBA, providing insights into key steps in HCCS mechanisms as well. We propose how release of holocyt c from cyt c synthases is induced by heme attachment and weakening of interactions of heme with specific, conserved residues at the active site. This weakening is a fundamental ramification of spontaneous thioether formation, which creates heme distortion.

## 2. Materials and Methods

### 2.1. In Vitro and In Vivo Measurements for HCCS Activities and Heme Attachment

We have previously described in vitro reconstitutions with purified HCCS and purified CcsBA proteins [21,28]. Analysis of heme attachment by SDS-PAGE and spectrally have been published [16,37]. For measuring levels of cyt c produced in vivo with *E. coli* recombinant HCCS (WT and variants), we co-expressed with cyt c [16,33]. We then used a modified assay that separated soluble cyt c from membrane-bound HCCS by ultracentrifugation [21]. Cyt c in the ultracentrifugation supernatant was quantified spectrophotometrically at 550 nm under reducing conditions.

### 2.2. Structural Modeling

**Figure 1:**
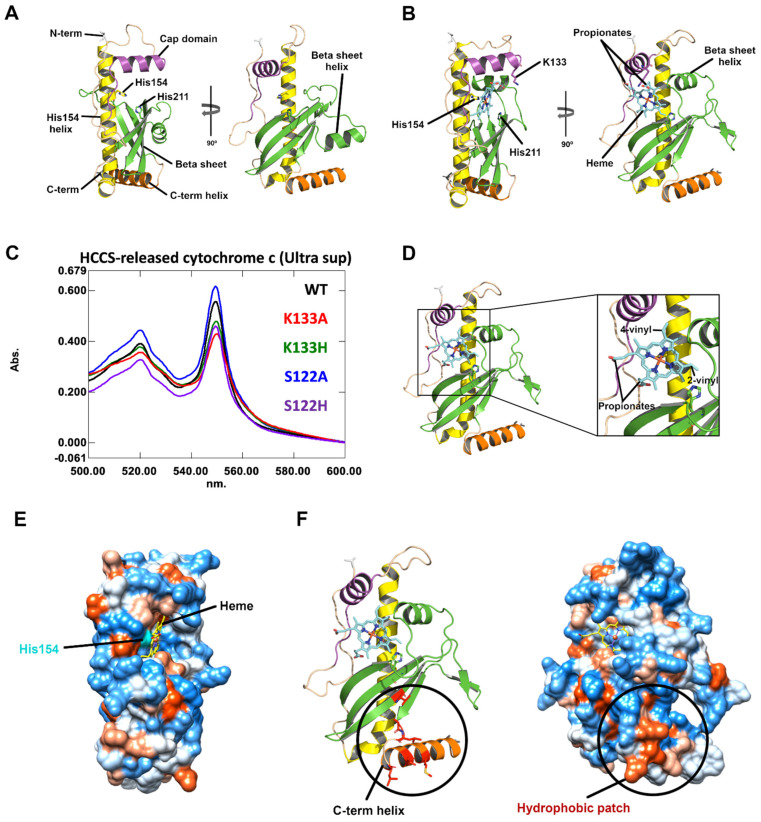
Human HCCS with and without heme. (**A**): AF3 model of human HCCS with newly designated domains: cap domain, magenta; His154 helix, yellow; beta sheet and beta sheet helix, green; C-term helix, orange. Axial ligand H154 and potential weak ligand H211 are labeled. PyMOL was used. (**B**): AF3 model of human HCCS bound to heme (cyan). K133 within the cap domain shown as stick (magenta). (**C**): A soluble release assay to quantitate levels of cyt c in *E. coli* recombinant ultracentrifuge supernatants. *E. coli* containing the IPTG-inducible HCCS and a second plasmid with an arabinose-inducible wt cyt c were used to determine HCCS activities (ability to produce soluble cyt c). WT HCCS and all HCCS variants are shown to biosynthesize and release substantial cyt c (550 nm) into the supernatant. (**D**): AF3 model of human HCCS bound to heme (cyan) with propionates rotated 90 degrees counterclockwise relative to its position in 1B to face hydrophilic environment. (**E**): Hydrophobicity surface of human HCCS with heme bound to active site, generated using UCSF Chimera. Hydrophobic regions in red, neutral regions in white, hydrophilic regions in blue. Heme in yellow, His154 surface in cyan. (**F**): Ribbon diagram of HCCS:heme with conserved residues V225, A248, L249, L252, V255, and M259 shown as red sticks on left. Hydrophobicity surface on right, generated using UCSF Chimera. A hydrophobic patch with conserved residues is circled. The patch may tether HCCS to the inner mitochondrial membrane.

Three-dimensional models of human HCCS, *S. cerevisiae* HCCS and HCC1S, *T. brucei* KCCS, N-terminus human cytochrome c and c1, and N-terminus *R. capsulatus* cytochrome c2 were generated by the AlphaFold3 server https://alphafoldserver.com/ (accessed on 28 April 2024) [35] for all figures except SFig3, where AlphaFold2 [34] and RoseTTAFold [36] were used instead. A structural model of myoglobin [Protein Data Bank (PDB) ID code 1MBN] was obtained from the Research Collaboratory for Structural Bioinformatics Protein Data Bank www.rcsb.org/pdb/home/home.do (accessed on 14 May 2024). Models for all ribbon structures were visualized and analyzed using PyMOL https://pymol.org/2/ (accessed on 28 April 2024), and hydrophobicity surface displays were generated and visualized using UCSF Chimera https://www.cgl.ucsf.edu/chimera/ (accessed on 14 September 2024). To generate a model of HCCS + heme with the heme propionates oriented outward facing the hydrophilic environment, the improperly oriented heme from the initial AF3 HCCS + heme model (as seen in Figure 1B) was replaced. A heme with the correct orientation, obtained from the AF3 HCCS + heme + cyt c output, was substituted to ensure that heme remained within the same plane while achieving the desired 90-degree rotation.

*S. cerevisiae* HCCS and HCC1S were truncated from N-termini to the first residues within the cap domain, W78 and W98, to confirm structural similarity to human HCCS. In *T. brucei* KCCS, residues extending from the N-terminus to M64 were truncated to maximize the per-residue model confidence score (pLDDT) assessed by AlphaFold3. pLDDT scores range from 0 to 100, with higher scores indicating increasing structural confidence. Truncated KCCS resulted in “high” (90 > pLDDT > 70) and “very high” (pLDDT > 90) scores. Residues downstream of I274 were also truncated due to low conservation between residues of KCCS and HCCS following the beta helix domain.

### 2.3. TM-Score for Assessing Truncation Point

The TM-score was used to assess the topological similarity between protein structures [38]. By weighing smaller-distance errors more strongly than errors of larger distances, TM-score value is more sensitive to the global structural similarity between two proteins than using RMSD, another commonly used measure for structural similarity. Additionally, the magnitude of TM-score is length-independent for random structure pairs, which makes for a more accurate measurement than RMSD and other measures that utilize length-dependent metrics. To determine the TM-scores used in SFig3, full-length FASTA protein sequences of human HCCS were submitted into AlphaFold2 and RoseTTAFold to produce the respective structures in PDB file format. Resulting PDBs were submitted as Structures 1 and 2 into a TM-score-determining server by the Zhang Lab (https://zhanggroup.org/TM-score/, accessed on 28 April 2024), providing a TM-score that indicated structural similarity on a scale from (0, 1]. Scores greater than 0.5 correspond to roughly the same fold, and a score of 1 reveals a perfect match between structures. Next, FASTA sequences were truncated from the N-terminus downstream to three separate residues (S52, A99, W118), submitted into AlphaFold2 (v1.5.5) and RoseTTAFold, and entered into the TM-score server to evaluate structural similarity at varying points of truncation. Truncation from the N-terminus to A99 of HCCS yielded the highest TM-score and was thus used as the sequence for developing the structural models used in this paper.

### 2.4. Video of HCCS in Its Activity Cycle

A molecular movie was made using UCSF Chimera version 1.17 (https://www.cgl.ucsf.edu/chimerax/index.html, accessed on 28 April 2024). Structures of HCCS in differing conformations were obtained via AF3, modeling heme with and without cyt c. Structure of cyt c was obtained from the PDB (ID: 3ZCF), and putative unfolded state was generated in PyMOL. Conformations were generated using the Chimera Morph Conformations feature.

### 2.5. Alignments of Primary Sequences of HCCS and KCCS

The protein sequences of HCCS in the eight diverse eukaryotic phyla were aligned by ClustalW [39], as seen in Supplementary Figure S1A. Residues were highlighted according to level of conservation, with dark blue indicating full conservation and light blue indicating partial conservation. In Supplementary Figure S1B, a new multiple-sequence alignment was generated by Clustal Omega [40] to incorporate three additional species of Kinetoplastids that utilize KCCS as opposed to HCCS. Jalview [41] was used to analyze the alignment of these eleven species, and the same method of shading was used in Supplementary Figure S1B as in Supplementary Figure S1A.

## 3. Results

### 3.1. Structures of Human HCCS, With and Without Heme

The HCCS enzyme in the mitochondrial intermembrane space has so far been refractile to structural analysis by crystallography. Unlike the large bacterial integral membrane cyt c synthases, where cryo-EM has been achieved [28,32], cryo-EM is limited by the small size of HCCS (i.e., 268 residues for human HCCS). A structural framework for HCCS would be very useful to inform new and interpret previous biochemical and spectroscopic studies. We initially used AlphaFold 2 (AF2, [34]) and RoseTTAFold [36] on human HCCS to evaluate structural models (Supplementary Figure S3). Both programs predicted N-termini with low confidence, implying that these regions are likely disordered. Although the mitochondrial targeting sequence of yeast HCCS was previously suggested to be uniquely internal to the protein [42,43,44], it is feasible that the N-terminus is a disordered target sequence. We truncated human HCCS at residue S52 and A99, repeating the predictions (Supplementary Figure S3). The A99 truncation resulted in a model of higher confidence, and both AF2 and RoseTTAfold yielded very similar HCCS structures, as shown by a template modeling (TM) score of 0.76. Further truncation (e.g., to W118) did not improve the TM-score. As shown in Supplementary Figure S1A, there is no primary sequence conservation up to residue A99. We used the A99 truncation for future analyses here, using the recent AlphaFold 3 (AF3, [35]), since it yielded nearly the same HCCS structure as AF2 (TM 0.98) but could be additionally used for substrate docking. 

We previously defined four domains in HCCS based on conserved residues when combined with mutational studies [3,18]. The AF3 structure is consistent with this (Figure 1A), whereby the “cap domain” (magenta) is domain I, the “His154 helix” (yellow) is domain II, and domains III and IV represent a “beta sheet” (green). Note the presence of a conserved, short alpha helix embedded in the beta sheet (“beta sheet helix”). We propose use of the new domain terminology based on the AF3 structures. As described below, these domain designations also infer functional relevance from biochemical and spectroscopic results. When comparing *S. cerevisiae* HCCS and HCC1S, we note that the cap domain, H154 helix, and beta sheet are each present (Supplementary Figure S4).

In using AF3 to model HCCS with heme (Figure 1B), we made two observations that required further analysis. The first was that the beta sheet helix was now located in an access point to the putative active site (i.e., an upward location). Below, we discuss this movement and its implication for heme access and protection. The second observation is that heme propionates were facing up towards the cap domain, and thus heme orientation changed by rotation of 90 degrees compared to when we included cyt c in the modeling. We used both AF3 and AutoDock Vina [45] to dock heme into HCCS with the cyt c substrate (Supplementary Figure S5). We constrained AutoDock Vina to use His154 as an axial ligand but did not use constraints for AF3. As shown in Supplementary Figure S5, heme was positioned by both programs within the same cavity and His154 as an axial ligand to the heme iron. AutoDock Vina tilted the heme slightly on the porphyrin edge where the vinyls are present (relative to the AF3 docked heme). In the AF3 structure, cyt c approximates the location where the beta sheet helix was in the HCCS structure alone. 

Thus, upon inclusion of the cyt c (16 or 20 mer) substrate with HCCS and heme in AF3 modeling, the heme is rotated approximately 90 degrees (Supplementary Figures S5 and S6), where the propionates are more exposed to the external hydrophilic environment. Based on similarity in positioning of heme using AutoDock Vina, and on vinyl locations with respect to thiols (of CXXCH, see below), we suggest that this heme orientation is more likely (than with the AF3model with only HCCS and heme—Figure 1B). Very recently, AF3 was used in a bioRxiv manuscript for *Plasmodium falciparum* HCCS with heme and cyt c [46]. In this structure, conserved K133 (human HCCS numbering) is located as a salt bridge to a heme propionate, 2–4 Å apart. K133 in our AF3 model of human HCCS with heme is also close to the propionates (Figure 1B). We tested whether cap domain residues K133 and S122 variants were required for recombinant human HCCS activity (Figure 1C). The activity assay measures spectroscopically the levels of cyt c synthesized and released into the *E. coli* soluble supernatant fraction. All HCCS cyt c products had absorption maxima of 550 nm, characteristic of cyt c attached heme. K133A and K133H variants, as well as S122 variants, all show nearly wild-type function in recombinant *E. coli* (Figure 1C and Supplementary Table S1). These results indicate that K133 is unlikely to form a required salt bridge to propionates. We suggest that the AF3 model of heme orientation is correct when the cyt c substrate is present but incorrect when only heme is included. Figure 1D shows the correct heme orientation in the HCCS AF3 model, whereby the beta sheet helix is in the up position. 

Figure 1E shows that heme would have access for entry adjacent to the cap domain (magenta) and into the cavity where His154 resides, with the beta sheet helix occluding access from the opposite side. We have shown previously [16] that HCCS His154A/G variants can be complemented for cyt c synthase activity by adding exogenous imidazole to recombinant *E. coli*. Presence of the strong His154 axial ligand in the buried cavity (Figure 1E) is consistent with this complementation. One interesting observation of the AF3 model is the presence of His211 in the beta sheet, with His211 close to but not acting as the second axial ligand to heme (Figure 1B). We have previously mutated conserved His211 and shown that while it is not required for function in recombinant *E. coli*, it is reduced to 20–50% activity [16,18], depending on the substitution (Supplementary Table S1). It is possible that His211 acts as a second, weak axial ligand, but not essential ligand during the HCCS cycle of activity. Many spectroscopic results, including exogenous imidazole liganding to purified HCCS [16,17,19,33], show that the weak ligand can be easily replaced (e.g., by imidazole). Moreover, resonance Raman spectroscopy on HCCS complexes with cyt c variants (e.g., CXXCA, CXXAH) show that the WT HCCS has a mixture of five and six coordinates, with His154 as the strong coordinate [17] (see Supplementary Table S1 and footnotes for a summary of spectral results). In a conformational change during activity that we propose below, His211 could be involved as a weak ligand during the HCCS cycle, potentially favoring one conformation.

Two hydrophobic regions are of potential interest in HCCS. One resides in the C-terminal helix, adjoining the last beta strand, with the helix exhibiting six conserved residues that form a hydrophobic patch (Figure 1F). We hypothesize that this patch could tether HCCS to the inner mitochondrial membrane, and would explain why the recombinant HCCS is localized to *E. coli* membranes, requiring detergents for purification [16]. The other hydrophobic region is the cavity where heme resides. Residues that form the heme binding site are discussed below in relation to biochemical and spectroscopic studies in each domain.

### 3.2. Cytochrome c1 (cyt c1) as a Substrate for the Human HCCS, and the Role of cyt c/c1 Residues in Binding

**Figure 2:**
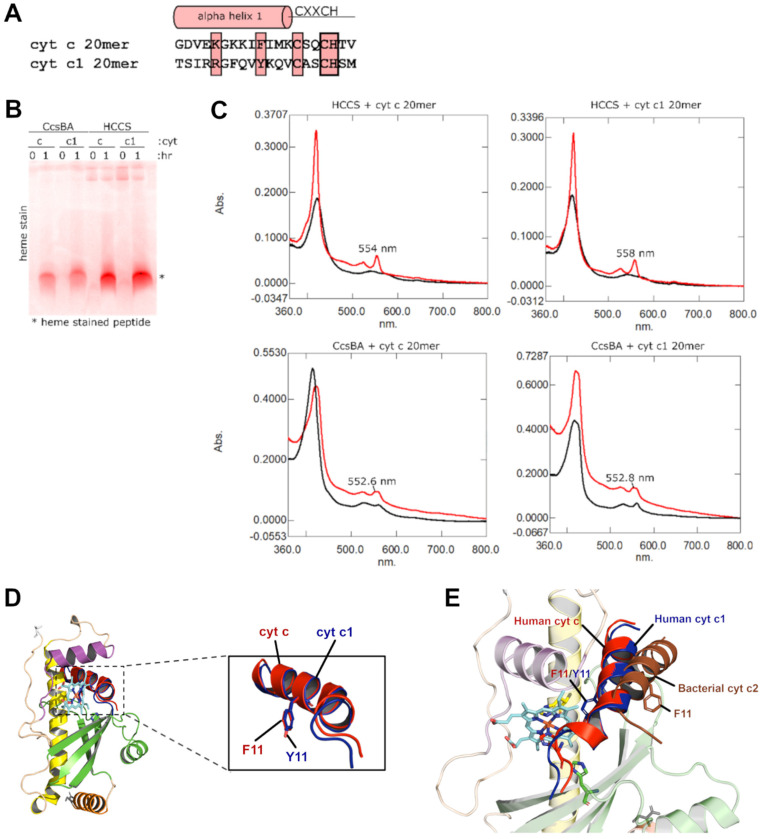
In vitro reconstitution analysis of cyt c_1_ as a human HCCS substrate. (**A**–**C**): HCCS and CcsBA attach heme to cyt c_1_ in vitro. (**A**): Sequences of two peptides used to assay the function of purified recombinant HCCS and CcsBA. Highlighted in pink are residues that have been shown to be important in cyt c recognition by HCCS. (**B**): Both HCCS and CcsBA react with the substrate peptide (cyt c or cyt c_1_) 20 mer ( incubated at 37 °C for 1 h with 1 mM DTT). Initial and final samples were run on SDS-PAGE gel, and show that the peptide is heme-attached after 1 h. Only heme covalently attached to the peptides is retained on the gel for heme staining. (**C**): UV–Vis spectra of HCCS/CcsBA and substrate peptides are shown before DTT addition (black) and after DTT and 1 h incubation at 37 °C (red). Spectral peaks at 554, 558, and 552 nm are consistent with heme attachment. (**D**): AF3 human HCCS, heme, 20 mer cyt c, and cyt c_1_ peptide docking. F11 of cyt c (red) and equivalent Y11 of cyt c_1_ (blue) shown as sticks in PyMOL. (**E**): 20mer human cyt c, cyt c_1_, and bacterial (*Rhodobacter capsulatus*) cyt c_2_ docked into human HCCS by AF3. Bacterial cyt c_2_ sequence (20 mer) is from *R. capsulatus*, with sequence GDAAKGEKEFNKCKTCHSI, as described previously.

We wanted to determine whether the cyt c_1_ substrate also requires the CXXCH motif and an N-terminal alpha helix, like cyt c. Unfortunately, recombinant studies co-expressing cyt c_1_ and the mitochondrial HCCS have not been successful, to our knowledge. This likely is due to the integral membrane properties of cyt c_1_ and assembly of the cyt b/c_1_ complex (complex III) after heme attachment to cyt c_1_. We have previously developed in vitro reconstitution using purified human HCCS, heme, and cyt c peptides [21]. A cyt c_1_ peptide of 20 residues (20 mer) was synthesized (Figure 2A) and we then determined whether it is recognized by HCCS for heme attachment. We used a cyt c 20 mer as a control, also determining whether the purified bacterial cyt c synthase (CcsBA) attaches heme to each peptide. CcsBA only appears to require the CXXCH motif for attachment [21]. Results were initially analyzed for attachment using SDS-PAGE, followed by heme staining for peptides that have attached heme (Figure 2B). Only peptides with covalently attached heme retain heme in these SDS gel systems. After 1 h incubations, both HCCS and CcsBA showed cyt c and cyt c_1_ peptides with heme (Figure 2B). Spectral analyses showed the emergence of absorption maxima characteristic of c-type heme after one hour of incubation (Figure 2C). As we have described previously for HCCS and cyt c [16,17], the appearance of sharp absorptions between 553 and 558nm is a property of HCCS/holocyt c complexes, and 550–552 nm are reflective of released cyt c. This is the case for products from both in vitro and in vivo studies. Thus, the spectral peak at 558 nm for cyt c_1_ suggests that it is not released from the HCCS active site, and that cyt c_1_ folding (as with cyt c) is part of the release mechanism (see next section). The bacterial CcsBA produced cyt c and c_1_ peptides with a lower maximum (Figure 2C, 552.8 nm), consistent with attachment and release. A second, slightly higher maximum for the CcsBA products can be attributed to the heme in CcsBA that remains in the TM-heme site, as we have discussed previously [21,28]. We conclude that purified human HCCS attaches heme to cyt c_1_ in vitro and that the CXXCH and upstream helix are recognized.

A structure of the human HCCS with heme and cyt c_1_ 20 mer was produced with AF3 (Figure 2D). The cyt c and cyt c_1_ peptides were positioned almost identically, including the histidine axial ligand (of CXXCH) and the two thiols positioned at the correct two vinyls of heme. Phe11 of cyt c has been a residue analyzed in past studies that appears to be important for HCCS recognition [16,20,23]. The Tyr of cyt c_1_ was positioned identically to the equivalent Phe11 of cyt c. The HCCS active site with heme and cyt c or cyt c_1_ shows that Phe/Tyr11 is positioned within the hydrophobic pocket directly above heme. The His154 helix has conserved hydrophobic residues (I149, I153, N157) on the same face of the helix as His154 that protrude into the hydrophobic cavity, interacting with the cyt c substrate (e.g., Phe/Tyr11).

We used AF3 to dock the bacterial cytc_2_ (a cyt c) to determine whether AF3 modeling would predict poor substrate interactions (Figure 2E). The bacterial cyt c_2_ is a very poor substrate for HCCS, and it has been shown previously by recombinant in vivo engineering and other approaches that a residue between Phe11 and Cys15 has been naturally deleted in evolution from the bacterial cyt c_2_ [20]. The bacterial cyt c synthases recognize only the CXXCH motif, so theoretically, no pressure from the perspective of assembly would occur to maintain the spacing in cyt c. Addition of this residue (i.e., Met13 in human cyt c) to cyt c_2_ makes the bacterial cyt c a good substrate for human HCCS in recombinant experiments. AF3 modeling (Figure 2E) showed very poor interaction of the bacterial cyt c_2_ 20 mer (sequence GDAAKGEKEFNKCKTCHSI) with HCCS and heme. Indeed, the equivalent residue to Phe11 of the bacterial cyt c is now facing outward, no longer in the hydrophobic cavity (Figure 2E). These results provide support for the AF3 models on HCCS. They also support the in vivo and in vitro analyses, showing that the helix adjacent to CXXCH in cyt c and cyt c_1_ are important for HCCS recognition.

We conclude that in vitro reconstitution of purified human HCCS with heme and cyt c_1_ peptides is a valid approach to study attachment and that the human HCCS active site exhibits similar recognition requirements for cyt c and cyt c_1_.

### 3.3. Structures of HCCS with Heme and cyt c Substrates: Functions of Conserved Residues and Domains Based on In Vivo Activities and Biochemical Analyses of Purified HCCS Complexes

**Figure 3:**
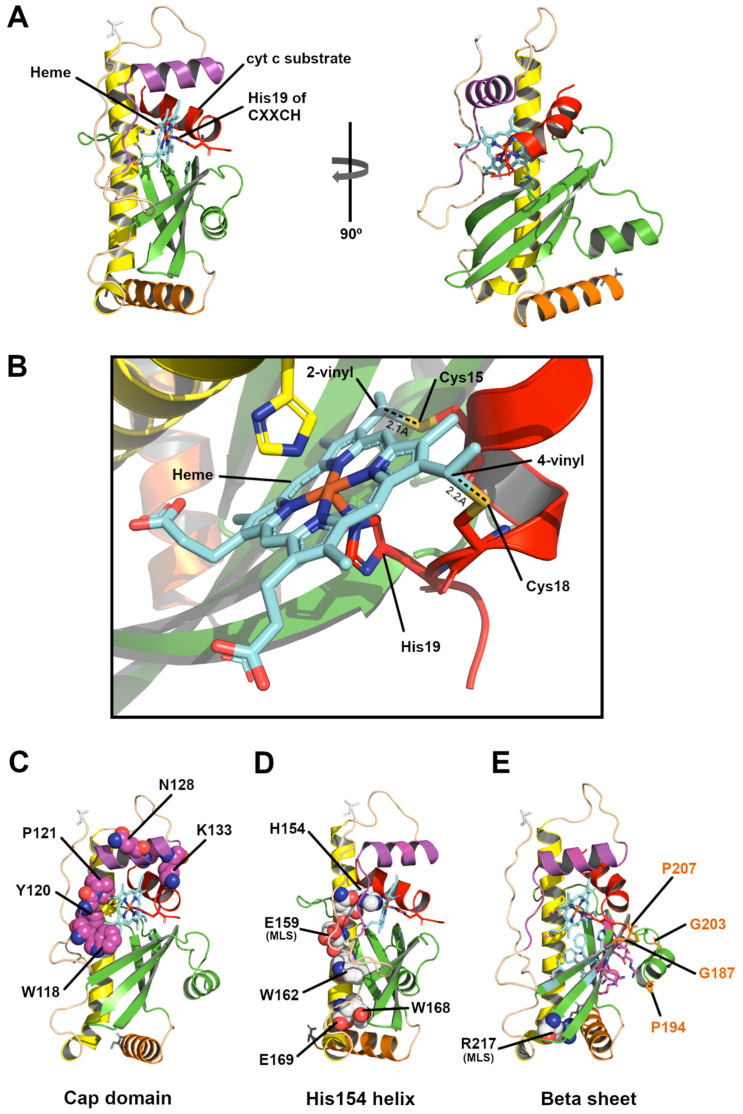
Structures of human HCCS with substrates heme and cyt c, delineating key residues involved in mechanisms and disease. (**A**): AF3 human HCCS, heme, and 16 mer human cyt c substrate (red) modeled using PyMOL. His154 of HCCS and H19 of CXXCH motif form axial ligands to heme. (**B**): Enlargement of active site where 2-vinyl and 4-vinyl groups of heme form thioether bonds with corresponding C15 and C18 of CXXCH motif. Distances between respective vinyls and thiols denoted with dashed lines. (**C**–**E**): Conserved residues that reside in the (**C**) cap domain, (**D**) H154 helix, and (**E**) beta sheet are depicted. Many of the variants with these conserved residues mutated have been studied biochemically and spectroscopically (see Supplementary Table S1), as discussed in the text. (**E**): Conserved residues within beta sheet shown as sticks either interacting with heme (cyan) or facing away from heme binding site (magenta). Residues that form the beta sheet helix (green) interact with cyt c when up: see Supplementary Figure S6 on how this helix is predicted to move in the activity cycle of HCCS.

We have shown previously that heme likely binds as a prerequisite to binding the second substrate, cyt c. For example, an HCCS (His154A) variant does not bind the cyt c substrate [16], as established by an inability to co-purify cyt c with HCCS (His154A). The same approach was used to show that CXXCH (WT), CXXAH, AXXCH, and CXXCA variants of cyt c co-purified with WT HCCS [17,19]. These results indicate that no individual cysteine (or histidine) in cyt c is essential for binding the cyt c substrate. Would AF3 dock cyt c such that the vinyls of heme are regio-specifically positioned near the proper cysteines? Using AF3, we docked heme into HCCS, then the cyt c 16mer (Supplementary Figure S6). When the cyt c 16 mer is docked with heme in HCCS, the beta sheet helix (green) moves down to interact with the outside face of the sheet, while the cyt c takes its place (Figure 3A; Supplementary Figure S6C). In this complex, His154 remains an axial ligand to the heme, but the second axial ligand is formed by histidine from the cyt c substrate (His19 of CXXCH) (Figure 3B). We have shown spectroscopically by co-purifications of many cyt c variants with HCCS [17], and by in vitro reconstitutions with cyt c peptides [21], that the cyt c histidine acts as the second axial ligand in the complexes. Resonance Raman spectroscopy has confirmed these structural features of the HCCS/cyt c complexes [17]. Importantly, the AF3 structure shows that the first cys thiol of CXXCH is 2.1 Å and the second cys thiol is 2.2 Å from the correct vinyls to form thioether bonds (Figure 3B). 

We have further analyzed the AF3 structures in the context of biochemical and spectroscopic results with HCCS WT and variants, dissecting the roles and positioning of conserved residues. Figure 3C displays the HCCS residues in the cap domain that have been mutated and studied for activity and spectral signatures of the purified variants (with and without cyt c bound). Some mutations in the conserved residues in the cap domain perturb the heme spectrally (Supplementary Table S1). We suggest that these heme spectral perturbations provide support for the AF3 structure, as each of these residues surrounds the heme. We designate this the “cap domain” because it appears to cap the heme, with residues located in the cap domain alpha helix and the loop adjacent to the putative heme entry point. Interestingly, although conserved, no single substitution in the cap domain appreciably impacted the ability to attach heme to cyt c (see “activity”; Supplementary Table S1). W118A HCCS is designated a “release mutant” and has higher activity than WT HCCS. We have proposed that these “release” variants increase the yield of cyt c by improved release of product holocyt c, weakening the interaction of heme with HCCS [33]. HCCS (E159A) in the His154 helix is in the release class, and it too is adjacent to heme (Figure 3D). Thus, the AF3 structures are consistent with biochemical results on these HCCS variants.

Figure 3D displays conserved HCCS residues in the His154 helix that have been studied mutationally and biochemically (Supplementary Table S1). A variety of studies cited above have shown that His154 acts as an essential axial ligand to heme in HCCS, and these will not be further discussed. An E159K substitution is one of two mutations linked to MLS, and recombinant studies on HCCS (E159K) have indeed shown it to possess low function [18], whereas HCCS (E159A) are release mutants with higher than WT activity. We suggest that these results support the AF3 structure, with this part of the helix adjacent to the heme and thus comprising part of the active site. Lower (C-terminal) in the His154 helix, W162 forms the base of the hydrophobic cavity/active site and is the first residue of His154 helix to interact with the beta sheet (Figure 3D). W168 and E169 of the His154 helix interact with the beta sheet and the C-terminal hydrophobic patch helix. Substitutions at W162, W168, and E169 in HCCS exhibit low activity and low yields of purified HCCS, suggesting stability issues (see Supplementary Table S1). Thus, the His154 helix is essential for heme binding and the structural integrity of HCCS, consistent with AF3 placing His154 as an axial ligand and “glue” residues that interact with other domains.

The conserved beta sheet (Supplementary Figure S1A) of HCCS forms the basin of the active site cavity where heme resides. Figure 3E displays all conserved sidechains of the beta sheet in stick format. Many sidechains (cyan) are facing the cavity and provide hydrophobic, direct interactions with heme. Surprisingly, many conserved sidechains (magenta) are on the opposite plane of the beta sheet facing away from the heme binding site. Other conserved residues form the beta sheet helix or an edge of the beta sheet that interacts with cyt c (Figure 3E). Cyt c binding would likely favor the conformation whereby the beta sheet helix interacts with the outer face of the beta sheet, and the beta sheet upper edge interacts with cyt c. We propose that two conformations predicted by AF3 are supported by the aforementioned dispersed locations of these conserved residues and by biochemical results. In one conformation, the beta sheet helix is located where cyt c will be (Supplementary Figure S6B), and a second conformation occurs upon cyt c binding, as shown in Figure 3E (and Supplementary Figure S6C). Four conserved residues that induce turns (Gly187, Pro194, Gly203, Pro207) form the hinges that toggle the beta sheet helix up and down (Figure 3E). Such toggling can also explain the conservation of residues on the outer face of the beta sheet, that is, for interaction when toggled down. Many of the substitutions in the HCCS beta sheet result in low activities and low yields of purified HCCS (Supplementary Table S1), suggesting that this domain provides integrity for stability or folding (e.g., formation of the active site). MLS substitution R217C is one such example of a “glue” residue (Figure 3E).

### 3.4. Release of Holocyt c from HCCS and CcsBA cyt c Synthases: Structural and Biochemical Considerations

**Figure 4:**
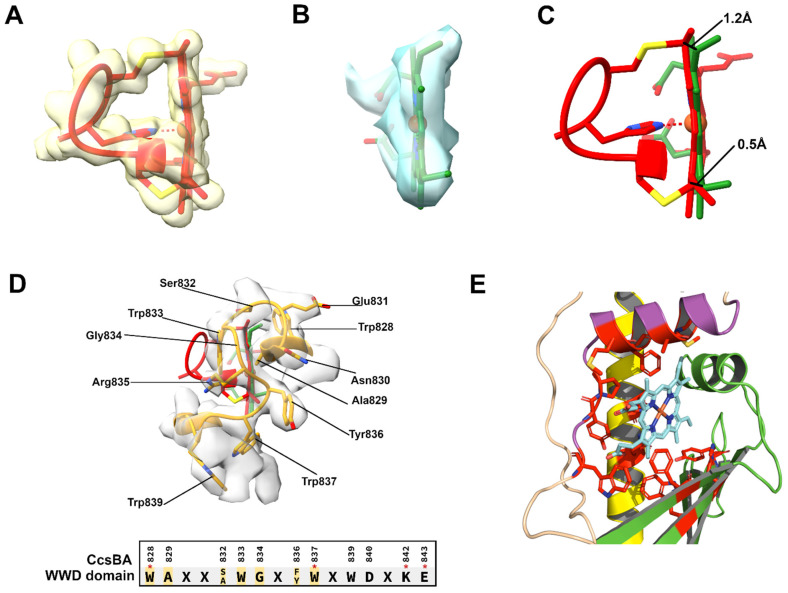
A structural basis of CXXCH attachment, leading to release of cyt c substrate: the bacterial cyt c synthase CcsBA active site from Cryo-EM. (**A**): Heme of crystal structure of human cyt c along with conserved CXXCH motif. Electron density from the crystallography present in transparent yellow. (**B**): Heme of Cryo-EM structure of *H. hepaticus* CcsBA shown in green. Cryo-EM electron density present in transparent blue. (**C**): Overlay of the experimentally determined structures of CXXCH and heme from human cyt c (red) along with b heme (green) at the CcsBA active site; distances between vinyl groups are shown. (**D**): Overlay of stick structures from (**A**,**B**), with Cryo-EM electron density of the WWD domain of CcsBA present in transparent grey. CcsBA residues that form a cys/heme crosslink are indicated with an asterisk. Conserved residues that contact heme are highlighted in yellow. (**E**): HCCS heme binding residues that are less than 2 Å from heme: cap domain: W118, Y120, P121, M125, F126, A129, M130; H154 domain: H154, N158; beta sheet: F185, H211, W213, Y228.

We previously proposed that two processes in the assembly of a native cyt c (and now cyt c_1_) are important for release of holocyt c from the HCCS active site [33]. The first is thioether formation (attachment), and the second is subsequent folding of cyt c. We based the first criterion in part on the distortion of heme created by two thioether bonds and the HCCS-mediated release properties of single and double thioethers in cyt c cysteine variants. Distortion was measured by resonance Raman spectroscopy and release from HCCS using various approaches [17]. Comparing c-heme in cyt c (Figure 4A) to b-heme (cryo-EM densities) at the CcsBA active site (Figure 4B), it is clear that the two thioethers in cyt c pucker or distort the heme from its planar geometry (Figure 4C). This has been known since crystal structures of cyt c were first solved [47,48]. It is generally agreed that covalent attachment provides stability to c-type cytochromes where their heme is never lost [49]. However, the reason for two thioethers (or consequent puckering) has remained a mystery. A typical distortion created by the thioether attachments is shown in Figure 4C, where one edge of heme is pulled 1.2 Å and the other 0.5 Å towards the cyt c (for reference, in the direction of the His ligand of CXXCH). 

To structurally determine what interactions in the cyt c synthase active site would be weakened by this thioether-based distortion, it is more realistic to use a cryo-EM structure of a cyt c synthase where b-heme is bound at the active site. The only cyt c synthase with such structures solved is the bacterial CcsBA, which in its “open” conformation has heme bound to the periplasmic active site [28]. We used this structure and analyzed heme interactions with the synthase that would be weakened upon distortion. One face of heme in CcsBA is liganded by P-His1, structurally and functionally analogous to His154 of HCCS. Adjacent to P-His1 in the structure is residue W828 (Figure 4D). Figure 4D shows seven residues (yellow highlights in WWD sequence) at the heme interface whereby puckering created by thioethers would weaken their interactions with heme. These seven residues are particularly relevant since they are part of the highly conserved “WWD domain” in bacterial cyt c synthases [37,50,51,52]. Each of the seven side chains is less than 2 Å from heme, and nearly all have densities that contact heme b. For example, the indoles of W828 and W833 stack with the heme pyrroles at the CcsBA active site and confer pi bonding interactions. Indeed, W828, at the pyrrole with 4-vinyl, crosslinks to the 4-vinyl group when substituted with a cysteine (W828C), and W837C to the 2-vinyl group [52]. We propose that movement of heme by approximately 1 Å, upon thioether formation, weakens binding of the holocyt c product at the active site, inducing release. Residues that interact with heme at the HCCS active site in the hydrophobic cavity would, by analogy to CcsBA, be weakened upon covalent attachment. For the bacterial cyt c synthases (both CcsBA and CcmF/H), it is clear that hundreds of different c-type cytochromes, including multiheme cyt c nanowires, likely depend solely on the CXXCH motif for both recognition and the release step. A mechanism for release based on thioether formation thus makes sense. Analyses of cryo-EM densities of the CcsBA WWD domain with heme substrate (Figure 4D) provide the structural basis for this release from the synthase. 

We performed a similar analysis to the HCCS structure with heme and CXXCH substrate, determining whether conserved residues are located near heme that might be weakened by thioether-based heme distortion. Figure 4E shows that thirteen sidechains are less than 2 Å from heme, ten of which are conserved or semi-conserved. These represent all three domains (cap domain: W118, Y120, P121, M125, F126, A129, M130; H154 domain: H154, N158; beta sheet: F185, H211, W213, Y228.) We suggest that weakening of these sidechain interactions with heme upon thioether formation are part of the release mechanism for HCCS.

### 3.5. Molecular Evolution of the Current Mitochondrial HCCS from the Mitochondrial Endosymbiont and the Kinetoplastid cyt c Synthase (KCCS)

Supplementary Figure S2 shows an evolutionary time line for HCCS, beginning with the ancestral endosymbiont approximately 1.2 billion years ago [3,53]. This endosymbiont was of alpha proteobacterial origin and thus had the System I cyt c biogenesis pathway, consistent with some mitochondria retaining System I. Until approximately 0.8 billion years ago, no diverged lineage possessed an HCCS (that is discoverable by primary sequence). Recently, the phylum of organisms called Euglenozoa (e.g., the class Kinetoplastidia) were bioinformatically analyzed [54] to determine what protein(s) they use to synthesize cyt c, a unique single thioether cyt c in their mitochondria (with a A/FAXCH motif). The synthase is called kinetoplastid cyt c synthase (KCCS), and it has some limited sequence homologies to HCCS, but not enough homology for successfully searching Euglenozoa genomes with HCCS (Supplementary Figure S1b). The KCCS gene from *Trypanosoma brucei* was overexpressed with the *T. brucei* cyt c in *E. coli*, and KCCS was able to attach heme to the cyt c, proving that it is the cyt c synthase [54].

**Figure 5:**
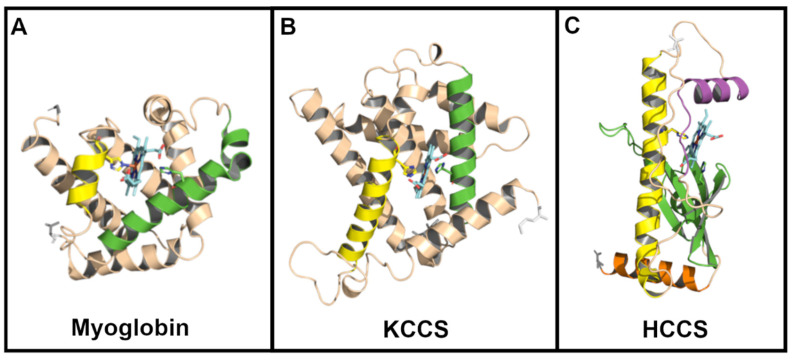
Hypothetical molecular evolution of the current day HCCS. (**A**–**C**): Structures show (**A**) myoglobin (1mnb, crystal structure), (**B**) KCCS (AF3-generated), and (**C**) human HCCS (AF3-generated). Protein identities and corresponding species are as follows**:** myoglobin, *P. catodon* (RSCB PDB ID: 1MBN); KCCS, *T. brucei* (NCBI XP_843981.1); HCCS, *H. sapiens* (Uniprot P53701). Heme is liganded in each case by histidines, as shown. Flow from (**A**–**C**) is a theoretical path by which a potential globin could gain heme attachment (to cyt c) activities through time, with Supplementary Figure S2 showing a taxonomic evolutionary tree.

To investigate whether there are similar structural features of KCCS to HCCS, we performed AF3 modeling on KCCS. We truncated the KCCS N-terminus, likely the disordered mitochondrial targeting sequence, to obtain a structure of high confidence. Figure 5B displays a helix (yellow) that contains the equivalent of His154 in HCCS, both structurally in the helix and by primary sequence analyses (Supplementary Figure S1b). Heme was located in a similar position relative to the HCCS cavity, but the HCCS beta sheet is absent; instead, another alpha helix (green) is present, and it possesses a second His that is predicted as an axial ligand. Conceptually, the kinetoplastid cyt c can be envisioned as binding in the same fashion to KCCS as in HCCS, with proper positioning of the A/FAXCH motif. More studies like those carried out on human HCCS will be needed to resolve mechanisms in attachment and release.

A question that remains on KCCS and HCCS is the molecular evolution of the current HCCS, since neither is related structurally or by primary sequence to the bacterial Systems I and II. We speculate that a heme protein similar to a globin may have duplicated after endosymbiosis and evolved to recognize the endosymbiotic cyt c. We show the structure of myoglobin in Figure 5A, envisioning that while System I was still operating, the duplicated myoglobin-type protein evolved, binding to cyt c, functioning more like the extant KCCS. Further evolution of this “KCCS” occurred (post-divergence to the extant KCCS) to evolve the HCCS (Supplementary Figure S2). This includes replacement of the second ligand helix (in KCCS) with the HCCS beta sheet. As shown in Supplementary Figure S2, the intermediates on the way to HCCS (other than KCCS) may remain unknown, but continued basic analyses of HCCS and KCCS could shed light on mechanisms and requirements. These would enlighten further hypotheses on the molecular evolution of HCCS.

## 4. Discussion

We describe a structural and biochemical basis for mitochondrial cyt c synthesis by HCCS. AF3 structures are consistent with what is known biochemically and spectroscopically of the human HCCS. In the attached video (Video S1), we show the cycle of HCCS activity starting with heme binding, cyt c binding, thioether formation, and release. Cyt c release from HCCS is stimulated by heme distortion and folding of cyt c. Studies [55,56,57] on cyt c folding have suggested that an early step includes interaction of the C-terminal helix with the N-terminal helix (adjacent to CXXCH). The N-terminal helix is known from in vivo [16,20,24,25,26] and in vitro [21] studies to be required for recognition by HCCS, consistent with the AF3 structural models. The video shows the N-terminal/C-terminal interaction in the release mechanism. Also shown in the video is protection of the active site by the beta sheet helix (see Supplementary Figure S6B). This potential protection by major conformational changes is functionally analogous to the bacterial cyt c synthase CcsBA, for which multiple cryo-EM structures have captured this dynamic process [28]. In CcsBA, opening and closing of a large periplasmic chamber (or a smaller beta sheet with some CcsBA proteins [32]) protects the active site, controlling access of the cyt c substrate.

The cryo-EM densities of CcsBA at the active site have been evaluated for insights on the release step of the holocyt c product (Figure 4). Stacking of conserved tryptophans with the pyrroles of heme, as well as other active site residues of the highly conserved WWD domain, would be disrupted upon thioether formation (Figure 4D). We thus suggest a structural basis for the release step based on puckering (distortion) that results from heme attachment. A very interesting analogy of cyt c heme release in cyt c synthases is to the enzyme ferrochelatase, which releases heme after iron is inserted into protoporphyrin. Release mechanisms in ferrochelatase have been analyzed for almost twenty years, in part based on structures and specific residues at the active site [58,59,60]. For example, a conserved pi helix at the active site undergoes conformational changes during the cycle of activity, and is involved in opening for release [58,61]. Iron insertion requires deprotonation, and this deprotonation upon iron insertion may induce pi helix movement [58,59,62]. Additionally, upon binding the substrate protoporphyrin, distortion occurs, but after iron insertion the heme product becomes more planar [60,63]. It has been proposed [60] that this change in planar geometry weakens interaction with heme and is thus part of the release mechanism.

Insights and cautions have been learned with regard to using AF3 for substrate docking and protein structures of HCCS. We discovered that heme is rotated depending on whether the cyt c substrate is included in AF3 modeling (Supplementary Figure S6). When cyt c is not included in the AF3 submission (i.e., with human HCCS and only heme), the heme is rotated such that a propionate is closer to K133 (Figure 1B). We provide evidence that K133 variants exhibit nearly wild-type function, in part leading us to suggest that AF3 has improperly rotated the heme. Indeed, when the cyt c substrate is included in the AF3 submission, heme is properly rotated such that vinyl groups are perfectly positioned to thiols of CXXCH for thioether attachment (Figure 3B, Supplementary Figure S6). The results point to caution necessary when using AF3 for substrate docking, as it is unlikely that heme rotates at the active site. Confirmation by biochemical, structural, or spectroscopic results remains important in modeling. 

The insights gained from AF3 structures are significant, as described in the results and visualized in Video S1. AF3 models may also assist in preparing HCCS proteins that can be structurally determined. For example, removing the N-terminal disordered regions and engineering the hydrophobic C-terminal helix (i.e., hydrophobic patch, Figure 1F) may increase solubility and stability. The AF3 models presented here, in conjunction with spectral and biochemical results, lead to well-defined hypotheses on the HCCS cycle that can be tested experimentally.

## 5. Conclusions

This study places biochemical and spectral studies into a structural framework for the mitochondrial cyt c synthase, HCCS. Previously, a four-step mechanism by HCCS has been proposed. Using AF3, the structural basis is described for heme binding (step 1), apocyt c binding (step 2), thioether attachment (step 3), and release of cyt c product (step 4). It is proposed that the active site heme is protected by a short alpha helix (called beta sheet helix) that toggles up and down: up when heme is present, down when the cyt c CXXCH substrate binds. An analogy is made to the bacterial cyt c synthase CcsBA, which uses a protective chamber or cap; the chamber is only “open” when the heme is present, so only then does substrate (CXXCH) bind. In the case of HCCS, we propose that heme is protected and that cyt c binding replaces the beta sheet alpha helix, with subsequent spontaneous thioether attachment. A universal mechanism for release of cyt c product is suggested. That is, thioether-based distortion of heme weakens many highly conserved heme-binding residues in both CcsBA and HCCS. This results in cyt c release and positioning of the cyt c synthase active sites for another round of attachment.

## Data Availability

All data are contained within the manuscript.

## Supplementary Materials

The following supporting information can be downloaded at: https://www.mdpi.com/article/10.3390/biom14121483/s1, Table S1: Analyses of conserved residues in human HCCS; Figure S1: Holocytochrome c synthase (HCCS) and kinetoplastid cytochrome c synthase (KCCS) protein sequence alignments; Figure S2: Evolutionary tree illustrating how pathways for cytochrome c biogenesis are distributed across various eukaryotic kingdoms; Figure S3: Structural similarity between human HCCS models by AlphaFold2 and RoseTTAFold; Figure S4: Comparison between *S. cerevisiae* HCCS, HCC1S, and human HCCS structures; Figure S5: Comparison of human HCCS, heme, and cyt c docking output using AutoDock Vina vs AF3; Figure S6: Mechanisms underlying human cyt c biogenesis by HCCS generated with AF3 models of HCCS + heme + cyt c sequentially; Video S1: Cycle of action of HCCS with cyt c folding and release.
