# Supplementary material for: Structural Insights into Mechanisms Underlying Mitochondrial and Bacterial Cytochrome c Synthases
Source: Biomolecules. 2024 Nov 21;14(12):1483. doi: 10.3390/biom14121483 (PMC11727520; doi:10.3390/biom14121483)
Supplement: Supplementary file 1 [file biomolecules-14-01483-s001.zip › biomolecules-3300986-supplementary-re/2024 Childs et al Supplemental Biomolecules REVISION Nov 12.pdf]

Supporting Information

Structural insights into mechanisms underlying mitochondrial and bacterial cytochrome c synthases.

Pema L. Childs, Ethan P. Lowder, Deanna L. Mendez, Shalon E. Babbitt, Amidala Martinie, Jonathan Q. Huynh, Robert G. Kranz*

Department of Biology
Washington University
St. Louis, MO 63146

Contents:

Supp Table 1. Analyses of conserved residues in human HCCS

SuppFig1: Holocytochrome c synthase (HCCS) and kinetoplastid cytochrome c synthase (KCCS) protein sequence alignments.

SuppFig2: Evolutionary tree illustrating how pathways for cytochrome c biogenesis are distributed across various eukaryotic kingdoms.

SuppFig3: Structural similarity between human HCCS models by AlphaFold2 and RoseTTAFold.

SuppFig4: Comparison between *S. cerevisiae* HCCS, HCC1S, and human HCCS structures.

SuppFig5: Comparison of human HCCS, heme, and cyt c docking output using Autodock Vina vs AF3.

SuppFig6: Mechanisms underlying human cyt c biogenesis by HCCS generated with AF3 models of HCCS + heme +cyt c sequentially.

Supp Video 1: Cycle of action of HCCS with cyt c folding and release**.**

Supp Table 1. Analyses of conserved residues in human HCCS.

New domain designation	Residue mutated (human HCCS)	Activity of mutant in *E.coli* (cyt c synthesis)[1]	Spectral properties of heme in purified HCCS variant[4.6]	Other information (e.g. disease)	Reference
**Cap domain** (previously referred to as Domain I)	W118	160%	Perturbation		Babbitt et al. 2014 *Biochem* (ref 18)
residues W118 to G134	Y120	70%	Perturbation		ref 18
	P121	90%	Perturbation		ref 18
	S122	S122A-100%<br>S122H-100%	like WT		This study[5]
	N128	80%	Perturbation		ref 18
	M130	90%	like WT		ref 18
	K133	K133A-70%<br>K133H-70%	like WT		This study[5]
**H154 helix** (residues Q144 to A174). (Previously Domain II)	H154	H154A-0%[3]<br>H154G-0%[3]<br>H154Y-0%	Perturbation: No heme binding		San Francisco et al 2013. *PNAS* (ref 16) and ref 18
	N155	190%	like WT		ref 18
	E159	E159A- 170%<br>E159K- 10%<br>E159D-140%	Perturbation (In E159A and E159K <50% steady state heme bound)	E159K is MLS mutation. E159A is a "release mutant"	ref 18
	W162	25%	ND (Low yield)		ref 18
	W168	10%	ND (Low yield)	part of the C-term hydrophobic patch	ref 18
	E169	E169A-8%<br>E169K-15%	ND (Low yield)		ref 18
**Beta sheet** (S181 to D250). Previously referred to as Domains III and IV	H211	H211A- 20%<br>H211G- 50%<br>H211Y-20%<br>H211C-20%	See "resonance Raman" summary on a potential second axial ligand[6]		ref 16, 18
	W213	120%	ND		ref 18
	R217	R217C-5%<br>R217A-10%<br>R217K-20%<br>R217D-15%	Low yield: Possible folding/stability defect	R217C is MLS mutation	ref 18
	D227	15%	ND		ref 18
	Y229	15%	ND		ref 18

	R246	20%	Low yield		ref 18
	D257	D257A-100%<br>D257K-25%	ND		ref 18
	R258	R258A- 15%<br>R258E-15%	ND		ref 18

Supp Table 1 Footnotes

1. Statistics provided in references. Mutations are to alanines unless stated.

2. Not determined.

3. Mutation is complemented for function by exogenous 10mM imidazole.

4. Spectra are typically of the purified HCCS variant with bound cyt c, as described in Babbitt et al. Biochem 2014-- ref 18. "Perturbation" indicates a significant change in spectral characteristics of reduced, oxidized Soret or alpha absorption peaks when compared to the WT HCCS/cyt c complex. In some cases, "perturbation" indicates a difference in levels of heme bound between the WT HCCS and the variant. We suggest that any perturbation (eg. with WT HCCS or WT HCCS/cyt c) indicates a close association of the residue with heme at the active site.

5 See SuppFig 7.

6. Information on HCCS and HCCS/cyt c complexes from visible spectra and resonance Raman spectra is summarized below.

<u>On the second axial ligand of heme in HCCS (the first being His154)</u>:

- **Exogenous imidazole acts as the second axial ligand (ie. 6th coordinate ligand) :** "*Soret maxima of the triple Cys/His mutant complex (Fig.3G) and HCCS alone (Fig. 3H) were both blue shifted 8–10 nm upon addition of imidazole. Thus the second axial ligand to heme in these two proteins can be provided by exogenous imidazole, thereby facilitating oxidation."* from Babbitt et al JBC 2014-ref 17.
- **rRaman spectra shows a mixture of 6c and 5c in the HCCS/cyt c His19 variant complexes: "***Note that the spectra of the complexes comprising theH19AandH19Mcyt c variants exhibit two v3 bands, one attributable to hexacoordinate (6c) LS heme at 1490 cm-1 and another at 1469 cm-1, typical of pentacoordinate (5c) high spin (HS) ferrous heme."* from Babbitt et al JBC 2014-ref 17.
- **Other rRaman spectral bands show a mixture of 6c and 5c: "***The bands in this frequency range of the spectra recorded from the His-19 variants show two bands at frequencies of ~1600 and 1581 cm-1, typical of 6c-LS and 5c-HS heme b, respectively."* from Babbitt et al JBC 2014-ref 17.

<u>On the puckering of heme (distortion)</u> when single or double thioethers are formed, analyzed using rRaman spectra: rRaman spectra was of HCCS/cyt c WT, C15 and C18 variant complexes. Data shows that the more cyt c released from HCCS, the more the puckering in the HCCS/cyt c complex.

- *"Interestingly, the single v48 frequency in the D4h system is split into two frequencies in theWT(632 and 643 cm&1) and C15S (631 and 647 cm&1), consistent with the aforementioned Cs porphyrin distortion. By contrast, v48 is activated in HCCS:heme:cyt c C18A, but splitting is not detected. This suggests diminished puckering of its 2,4-B-pyrrole edge relative to that imposed in the C15S complex. This, we hypothesize, has implications for the role of the residues at positions 15 and 18 in the assembly mechanism."* from Babbitt et al JBC 2014-ref 17.
- *In discussion: " The RR spectra of HCCS complexes with WT, C15S, and C18A cyts c indicate that the WT exhibits more puckering than C15S, and C15S exhibits more puckering than the C18A variant. This is consistent with the results that C15S is released (step 4) at ~8% WT levels and C18A at only 3% WT levels (12). We propose that puckering induced by spontaneous thioether bond formation reduces the interaction of heme with HCCS, leading to release (step 4)."* from Babbitt et al JBC 2014-ref 17.
- These spectral data are consistent with the biochemical release data (with variant peptides) published in Sutherland et al, eLIFE (ref 21).

SuppFig1: Holocytochrome c synthase (HCCS) and kinetoplastid cytochrome c synthase (KCCS) protein sequence alignments.

Supp Fig1A: HCCS sequences from eight diverse eukaryotic phyla are displayed with conserved residues colored in shades of blue to indicate conservation levels, similar to the CustalW alignment described by Babbitt et al.TIBS (2015)-- ref 3. Structural/functional domains are shaded analogous to coloring of human HCCS in previous figures. Yellow and green stars mark conserved H154 and H211 residues according to human HCCS sequencing. Grey arrow above A99 of human HCCS indicates the starting point for structure generation, and α-helix (Beta sheet helix) labeled within Beta sheet domain. Sequences with extensive gaps were condensed, and numbers enclosed by brackets represent removed residues for simplicity. Alignment was generated by ClustalW. Groups and corresponding species are as follows: Human HCCS, *Homo Sapiens* (UniProt P53701); Green alga HCCS*, Volvox carteri* (NCBI XP_002952003); Red alga HCCS, *Chondrus crispus* (NCBI XP_005713760); Choanoflagellate HCCS, *Monosiga brevicollis* (NCBI XP_001749606); Fungi HCCS, *Saccharomyces cerevisiae* (UniProt P06182); Amoeba HCCS, *Polysphondylium pallidum* (NCBI EFA80147); Apicomplexan HCCS, *Plasmodium falciparum* (UniProt Q8I5H1); and Phytoplankton HCCS, *Thalassiosira oceanica* (NCBI EJK64518).

Supp Fig1B: A protein sequence alignment of the existing eukaryotic phyla using HCCS in addition to various Euglenozoa KCCS (for cyt c biogenesis). His154 helix domain and star above conserved H154, yellow; Alpha helix domain and star above conserved H211, green. Alignment was generated by ClustalW and conservation was analyzed using Jalview. Euglenozoa sequences are as follows: *Trypanosoma brucei* KCCS (NCBI XP_843981.1), *Leishmania mexicana* KCCS (NCBI XP_003872427.1), *Trypanoplasma borreli* KCCS (BLAST analysis from GHOB01000228.1).

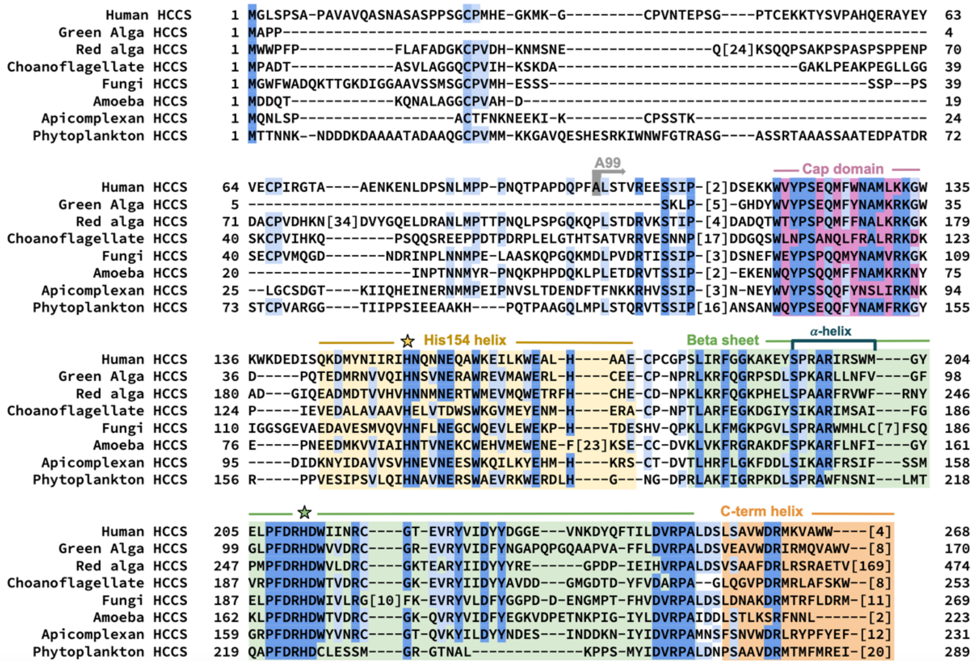

SFig1b - MSA with KCCS

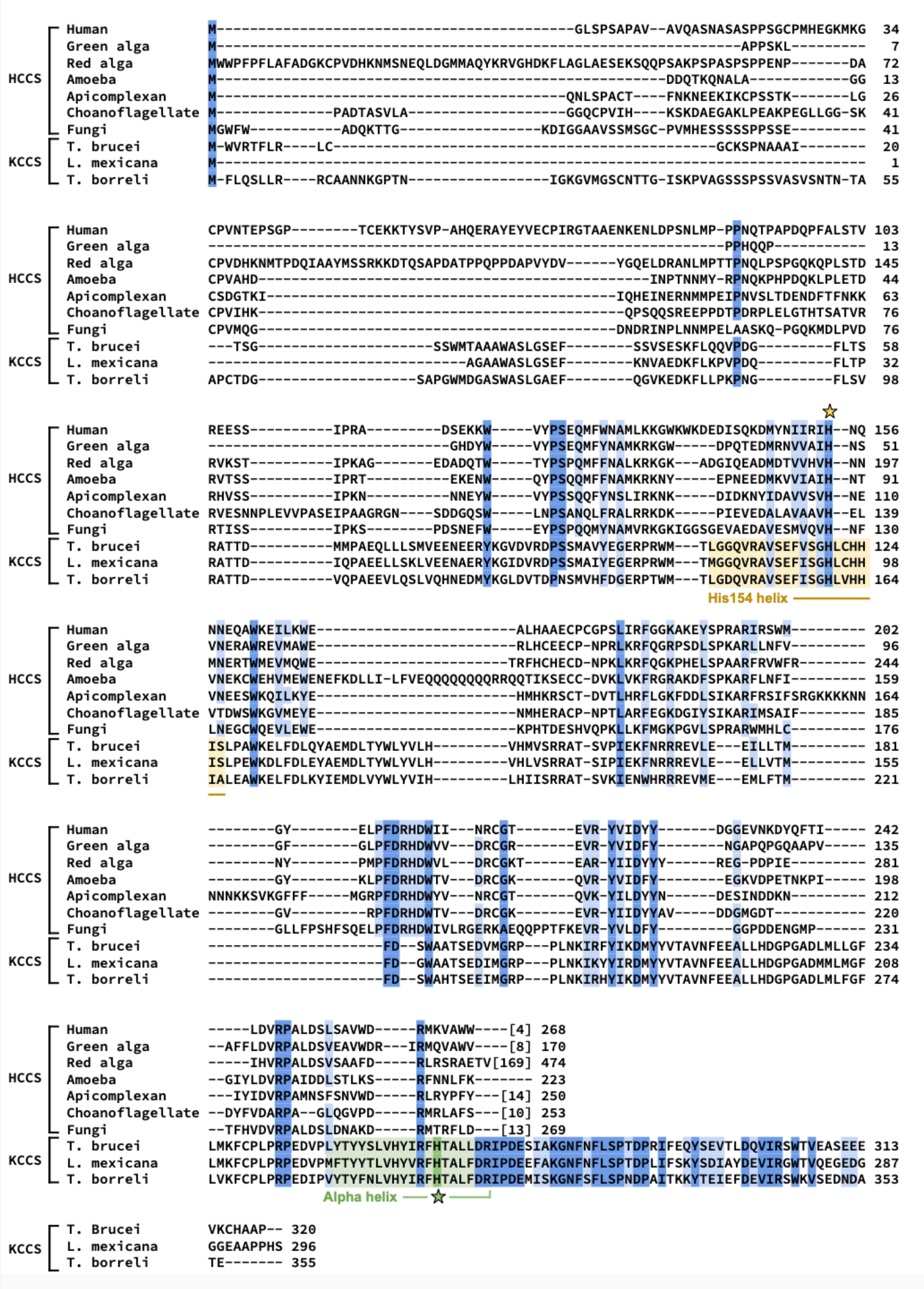

SuppFig2: Evolutionary tree illustrating how pathways for cytochrome c biogenesis are distributed across various eukaryotic kingdoms, similar to tree generated in Babbitt et al, TIBS, 2015-- ref 3. Those that utilize System I or System III are represented by (I) or (III: HCCS) following the name of the organism. Those that do not have either system due to a lack of mitochondria are denoted with 'no mito'. Organisms that use kinetoplastid cytochrome c synthase are denoted with 'KCCS'.

From top left to bottom right, the scientific names of organisms used are as follows: *Saccharomyces cerevisiae*, *Homo sapiens*, *Monosiga brevicollis*, *Capsapora owczarzaki*, *Entamoeba histolytica*, *Dictyostelium discoideum*, *Reticulomyxa filosa*, *Plasmodium falciparum*, *Tetrahymena thermophila*, *Thalassiosira oceanica*, *Dinenympha exilis*, *Giardia lamblia*, *Trichomonas vaginalis*, *Reclinomonas americana*, *Naegleria fowleri, Percolomonas cosmopolitus*, *Malawimonas jakobiformis*, *Cyanophora paradoxa*, *Arabidopsis thaliana*, *Chlamydomonas reinhardtii*, *Chondrus crispus*, *Cyanidioschyzon merolae*, *Trypanosoma brucei*, *Leishmania mexicana*, *Trypanoplasma borreli*. Abbreviation: bya (billion years ago).

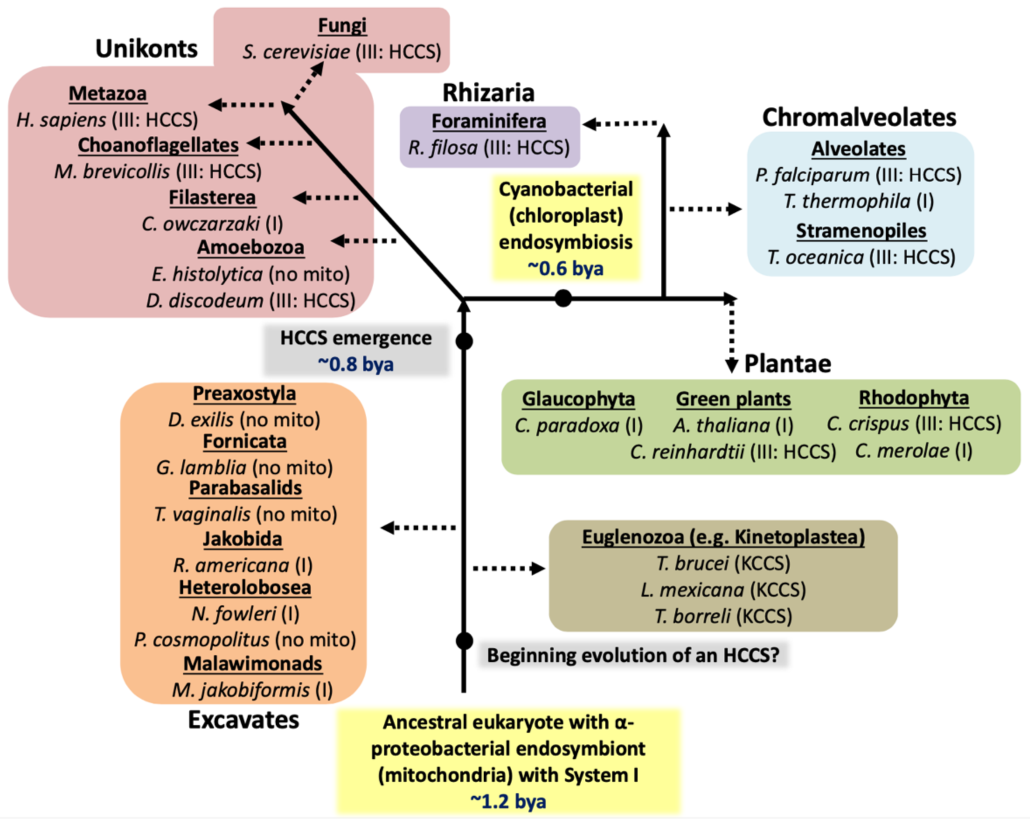

SuppFig3: Structural similarity between human HCCS models by AlphaFold2 and RoseTTAFold. Overlay of structures derived from AlphaFold2 (red) and RoseTTAFold (blue) with TM-scores (www.zhanggroup.org) indicating structural similarities. Truncation of N-term residues upstream of A99 yielded the highest TM-score, highlighted in green, so the A99 truncation was used in this study.

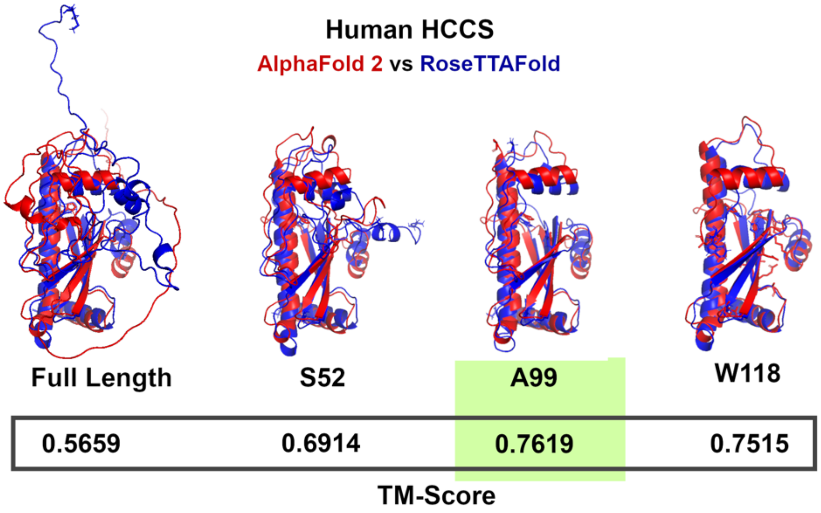

SuppFig4: Comparison between *S. cerevisiae* HCCS, HCC1S, and human HCCS structures.
A: AF3 models of *S. cerevisiae* HCCS and HCC1S superimposed indicate structural similarity among Cap domain, H154 helix, Beta sheet, and C-term domains. N-term truncations at W92 and W78 of ScHCCS and HCC1S, respectively, remove low-certainty disordered ribbon structures.
B: A99 human HCCS structure added to superimposed yeast (*S. cerevisiae*) HCCS and HCC1S. Varying loop structure within Beta sheet turn labeled according to protein identity: human HCCS (blue), yeast HCCS (red), yeast HCC1S (purple).

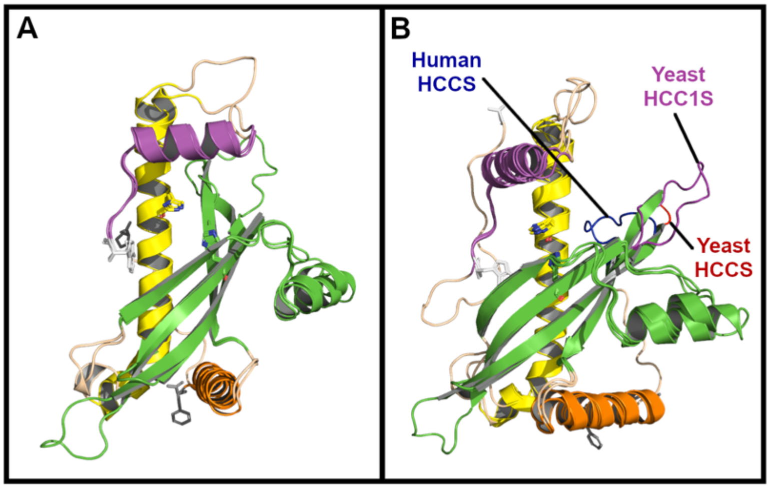

SuppFig5: Comparison of human HCCS, heme, and cyt c docking output using Autodock Vina vs AF3. Entire structures are displayed above respective boxes zoomed in on active site where heme and 16mer cyt c substrate bind. Both software position heme within the same cavity with H154 (yellow) as an axial ligand. Differences in “tilt” of heme result in reduced steric clash in the model generated by AF3 compared to Autodock Vina.

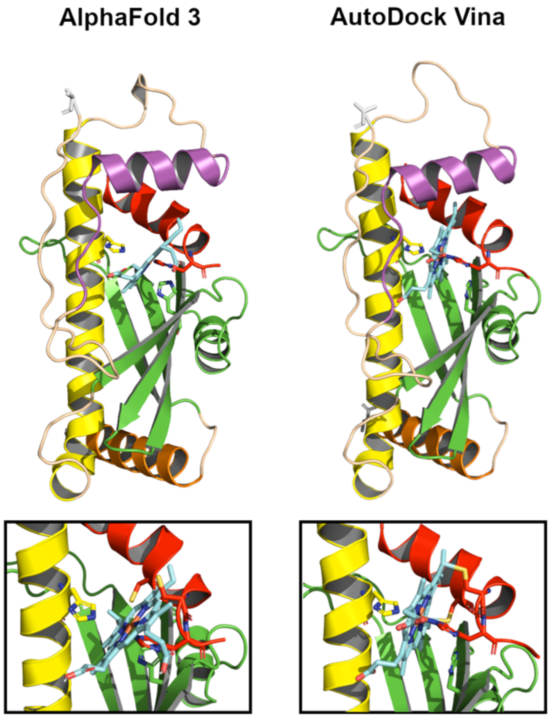

SuppFig6: Mechanism underlying human cyt c biogenesis by HCCS generated with AF3 models of HCCS + heme +cyt c sequentially.

Top panels display entire structures, bottom panels zoomed in on active site.

A: Human HCCS before heme binds. Region between H154 (yellow) and H211 (green) forms cavity for heme to enter.

B: Heme binds HCCS via axial ligand H154. Heme propionates face up towards Cap domain, and conformational change moves Beta sheet helix (green) in close proximity to heme.

C: 16mer cyt c substrate (red) binds heme of HCCS, and H19 of CXXCH forms second axial ligand to heme. Heme propionates rotate 90º (from "B") to face outside surface of HCCS into hydrophilic environment. Thioether bonds form between 2-vinyl and Cys15, and 4-vinyl and Cys18 of heme and CXXCH. Another conformational change returns Beta sheet helix (green) to original position nearer C-term helix, and interacting with the outer face of the Beta sheet.

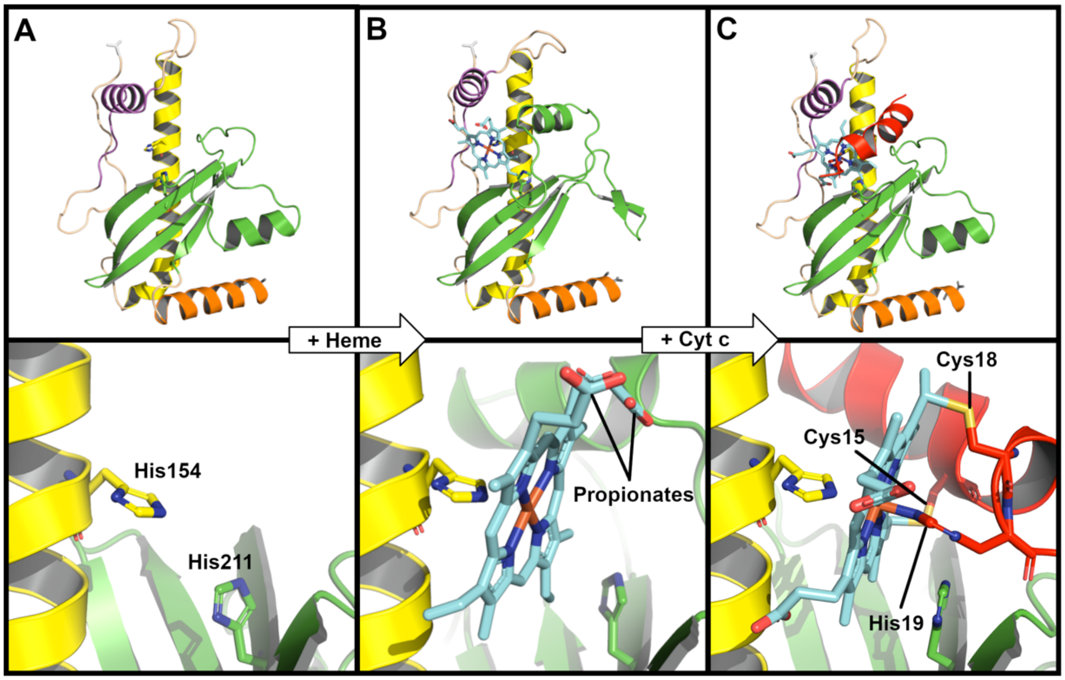

Supp Video 1: Cycle of action of HCCS with cyt c folding and release**.**

Human HCCS is shown with heme at the active site. Unfolded human cyt c approaches with both cysteines of its CXXCH motif (shown in sticks). As cyt c approaches, HCCS undergoes conformational change to allow the CXXCH region to bind the active site near the heme. Cysteines of CXXCH motif undergo spontaneous thioether attachment to the vinyl groups of heme. Cyt c begins to fold, inducing full release from HCCS, whereby the cyt c N-terminal and C-terminal helix interact first. Subsequent completion of folding to its native state is shown. Upon release, the Beta sheet alpha helix moves to its previous location, and a new heme enters the active site, poising HCCS to begin the cycle again.
